# Journal of Translational Medicine reviewer acknowledgement 2015

**DOI:** 10.1186/s12967-016-0818-8

**Published:** 2016-03-11

**Authors:** Francesco Marincola

**Affiliations:** Sidra Medical and Research Center, Doha, Qatar

## Reviewer acknowledgement

The Editors of *Journal of Translational Medicine* would like to thank all our reviewers who have contributed to the journal in Volume 13 (2015).

**Sayed Abdelwahab**

Egypt

**Ludovico Abenavoli**

Italy

**Jg Abreu**

Brazil

**Raffaele Addeo**

Italy

**Adetola Adesida**

Canada

**Sagheer Ahmed**

Brunei

**Wk Aicher**

Germany

**Laura Alaniz**

Argentina

**Donatella Aldinucci**

Italy

**Edoardo Alesse**

Italy

**H. Richard Alexander**

USA

**Jean-Pierre Allain**

UK

**Ahmed Al-Qahtani**

Saudi Arabia

**Frédéric Amant**

Belgium

**Ryszard Amarowicz**

Poland

**Muriel Amathonnet**

France

**Aditya Ambade**

USA

**Guoy An**

China

**Anuranjan Anand**

India

**Julian Ananiev**

Bulgaria

**Sarah Andres**

USA

**Polychronis Antonitsis**

Greece

**Vivekanandhan Aravindhan**

India

**Thurayya Arayssi**

Qatar

**Adria Arboix**

Spain

**Mario Arciello**

Italy

**Andressa Ardiani**

USA

**David Argyle**

UK

**Elif Damla Arisan**

Turkey

**Abdelilah Arredouani**

Qatar

**Inesa Arstikyte**

Lithuania

**Anders Asberg**

Norway

**Hitoshi Ashida**

Japan

**Stephanie Astrow**

USA

**Djordje Atanackovic**

Germany

**Djordje Atanackovic**

USA

**Stephen Atkin**

Qatar

**David Avigan**

USA

**Julio Ayala**

USA

**Angel Ayuso-Sacido**

Spain

**Ricardo Azziz**

USA

**Biersack B**

Germany

**Lorena Baccaglini**

USA

**Christina Bade-Doeding**

Germany

**Radja Badji**

Qatar

**Mohammed Badruddoja**

USA

**Peter Bai**

Hungary

**Salar Bakhtiyari**

Iran

**Ligia Bancu**

Romania

**Sanghamitra Bandyopadhyay**

India

**Barbara Barbaro**

Italy

**Maria Marcela Barrio**

Argentina

**Shashi Baruah**

India

**Giuseppina Basini**

Italy

**Purusotam Basnet**

Norway

**C.N. Baxevanis**

Greece

**Zeliha Bayram**

Turkey

**Fuller Bazer**

USA

**Nicole Beauchemin**

Canada

**George Beck**

USA

**Nicolau Beckmann**

Switzerland

**Diana Bell**

USA

**Giuseppe Bellisola**

Italy

**Gavin Bendle**

The Netherlands

**Armand Bensussan**

France

**Michael Bergmann**

Austria

**Catherine Berthomieu**

France

**Antonio Bertoletti**

Singapore

**Nicholas Bertos**

Canada

**Pascale Bertrand**

France

**Ricardo V Bessa-Nogueira**

Brazil

**Ali Bettaieb**

France

**Avinash Bhandoola**

USA

**Anshu Bhardwaj**

India

**Deepa Bhartiya**

India

**Karen Bieback**

Germany

**Carlo Bifulco**

USA

**Catherine Bisbal**

France

**Priscilla Biswas**

Italy

**Lena Björkman**

Sweden

**Julia Blanco**

Spain

**Jeronimo Blanco**

Spain

**Michael Blennerhassett**

Canada

**Javier Blesa**

Spain

**Erik Blok**

The Netherlands

**Edimar Bocchi**

Brazil

**Manfred Boehm**

USA

**Martin Bonamino**

Brazil

**R. Daniel Bonfil**

USA

**William Bonner**

USA

**Sahra Borges**

USA

**Laszlo Boros**

Hungary

**Francisco Borrego**

Spain

**Emanuela Bostjancic**

Slovenia

**Hamid Boulares**

USA

**Claudio Brancolini**

Italy

**Timo Brandenburger**

Germany

**Caterina Brasacchio**

Italy

**Gary Braun**

USA

**Caroline Breitbach**

USA

**Guilherme Bresciani**

Chile

**Nicola Brown**

Australia

**Joseph Bryant**

USA

**Ulrike Buchwald**

USA

**Alfredo Budillon**

Italy

**Linda Burkly**

USA

**Gregory Calip**

USA

**Sidia M. Callegari-Jacques**

Brazil

**Rafael Camargo**

Brazil

**Mark Cameron**

USA

**Robert Canter**

USA

**Jiatian Cao**

China

**Wei Cao**

China

**Haiming Cao**

USA

**Sergio Capaccioli**

Italy

**Emilia Caputo**

Italy

**Michele Caraglia**

Italy

**Nora Cardona-Castro**

Colombia

**Gloria Patricia Cardona-Gomez**

Colombia

**Anna Caretti**

Italy

**Ivana Carey**

UK

**Matteo Carlino**

Australia

**Tom¿ Carroll**

Ireland

**Dennis Carson**

USA

**Wayne Carver**

USA

**Luciano Castiello**

Italy

**Luciano Castiello**

Italy

**Patricia Castro Santos**

Spain

**Georgescu Ce**

Romania

**David Chafin**

USA

**Antonio Carlos Chagas**

Brazil

**Roger Chammas**

Brazil

**Stephen Chan**

USA

**Kw Chan**

Hong Kong

**Hsueh-Wei Chang**

Taiwan

**Wen-Wei Chang**

Taiwan

**Christopher Chang**

USA

**Jehad Charo**

Germany

**Tapas Kumar Chaudhuri**

India

**Damien Chaussabel**

Qatar

**Novel N. Chegou**

South Africa

**Fa-Ming Chen**

China

**Chunyan Chen**

China

**Chuan Chen**

China

**Hui Chen**

China

**Junqiang Chen**

China

**Lijuan Chen**

China

**Fuxue Chen**

China

**Bor-Sen Chen**

Taiwan

**Mei-Lien Chen**

Taiwan

**Zongming Eric Chen**

USA

**Chu-Huang Chen**

USA

**John Chen**

USA

**Ye Chen**

China

**Hao Cheng**

China

**Xi Cheng**

China

**Alfred Cheng**

Hong Kong

**Zhijie Jey Cheng**

USA

**Sung-Gil Chi**

South Korea

**Ya-Hui Chi**

Taiwan

**Maurizio Chiriva-Internati**

USA

**Miguel Chiurillo**

Venezuela

**Mi-La Cho**

Iammila@Catholic.Ac.Kr

**Mi-La Cho**

South Korea

**Dong-Sic Choi**

South Korea

**Murim Choi**

South Korea

**Eun Young Choi**

South Korea

**Mukund Chorghade**

USA

**Mukund Chorghade**

USA

**Becky Christian**

USA

**Georgios Christou**

Greece

**George Chrousos**

Greece

**Ji Chuang**

Taiwan, China

**June-Key Chung**

South Korea

**Christine Chung**

USA

**Dana Mihaela Ciobanu**

Romania

**Shiyuan Cjeng**

USA

**William Coleman**

USA

**Jucimara Colombo**

Brazil

**Begonya Comin-Anduix**

USA

**Angelo Giuseppe Condorelli**

Italy

**Jose Conejo-Garcia**

USA

**Costanza Conti**

Italy

**Leonel Cordoba**

Costa Rica

**Robin Cornelissen**

The Netherlands

**Andrea Cossarizza**

Italy

**Alexessander Couto Alves**

UK

**Johannes Coy**

Germany

**David Cram**

China

**Dragos Cretoiu**

Romania

**Lucio Crino’**

Italy

**Kylie Crompton**

Australia

**Bla¿ Cugmas**

Slovenia

**Chiara Cugno**

Qatar

**William Culp**

USA

**Giuseppe Curigliano**

Italy

**Brian Czerniecki**

USA

**Nayle Da Silva**

Brazil

**Agustin Dalmasso**

USA

**Bruno Daniele**

Italy

**Gennaro Daniele**

Italy

**Fabrice Danjou**

France

**Fida Dankar**

Qatar

**Nazir Dar**

India

**Piyali Dasgupta**

USA

**Jean Pascal De Bandt**

France

**Tjaart De Beer**

Switzerland

**Jana De Boniface**

Sweden

**Laura De Girolamo**

Italy

**Andrea De Maria**

Italy

**Valli De Re**

Italy

**Anita De Rossi**

Italy

**A. De Siervi**

Argentina

**Roel De Weger**

The Netherlands

**Olivier De Wever**

Belgium

**Julie Decock**

Qatar

**Maureen Deehan**

Switzerland

**Nuria Del Olmo**

Spain

**Bahman Delalat**

Australia

**Colleen Delaney**

USA

**Christian Delles**

UK

**Yanru Deng**

USA

**Melissa Derycke**

USA

**Sophie Desplat-Jégo**

France

**Massimo Di Maio**

Italy

**Stefania Di Marco**

Italy

**Laura Di Renzo**

Italy

**Sandra Diebold**

UK

**Michelle Digiacomo**

Australia

**Xiaoqiang Ding**

China

**Shaillay Dogra**

Singapore

**Kaoru Dohi**

Japan

**Riccardo Dolcetti**

Italy

**Helmut Dolznig**

Austria

**Pere Domingo**

Spain

**Shiwu Dong**

China

**Peixin Dong**

Japan

**Gina Doody**

UK

**Thilo Dork**

Germany

**Simon Dovedi**

UK

**Norman Dovichi**

USA

**Hans C. Dringenberg**

Canada

**Jaroslav Drsata**

Czech Republic

**Jun Du**

China

**Alfonso Duenas-Gonzalez**

USA

**Hélène Dumortier**

France

**Greg Dusting**

Australia

**Almac E.**

USA

**Sancak Eb.**

Turkey

**M. Eberl**

UK

**Jose Echevarria**

Spain

**Gunter Eckert**

Germany

**Ihab El Madhoun**

Qatar

**Bernice Elger**

Switzerland

**Hassan El-Sayyad**

Egypt

**N. Elsherbini**

Egypt

**Edward Emmott**

UK

**Elena Enache**

Romania

**Anna-Mart Engelbrecht**

South Africa

**Perenlei Enkhbaatar**

USA

**Amalia Enriquez-De-Salamanca**

Spain

**Alan Epstein**

USA

**Philipp Erben**

Germany

**Neta Erez**

Israel

**Mark Erlander**

USA

**Galileo Escobedo**

Mexico

**Xavier Estivill**

Spain

**Fabio Fabbian**

Italy

**Chiara Fabbro**

Italy

**Astrid V. Fahlenkamp**

Germany

**Birthe Fahrenkrog**

Belgium

**Stefano Fais**

Italy

**Domenick Falcone**

USA

**Xiaolong Fan**

Sweden

**Arghavan Farzadi**

Malaysia

**Todd Fehniger**

USA

**Ariel Feldstein**

USA

**Jane Ferguson**

USA

**David Ferland-Mccollough**

UK

**Cristiano Ferlini**

USA

**Silvia Martina Ferrari**

Italy

**Ludovic Ferretti**

France

**Clodoveo Ferri**

Italy

**Robert Ferris**

USA

**Fernando Fierro**

USA

**Fabiana Filigheddu**

Italy

**Brian Finck**

USA

**Ugo Fiocco**

Italy

**Andre Fischer**

Germany

**Timothy Fitzgerald**

USA

**Willy Flegel**

USA

**Megan Foeller**

USA

**Valentina Folgiero**

Italy

**Joseph Fontes**

USA

**Brian Ford**

UK

**Claudio Fozza**

Italy

**Gail Fraizer**

USA

**Renato Franco**

Italy

**Carlos Franco-Pparedes**

USA

**Vanessa Morais Freitas**

Brazil

**Marilia Freitas Calmon**

Brazil

**Gregory K. Friedman**

USA

**Jan Frystyk**

Denmark

**Jiao Fu**

China

**Yang-Xin Fu**

USA

**Dietmar Fuchs**

Austria

**Maria Fuciarelli**

Italy

**Shin-Ichiro Fujii**

Japan

**Shigetomo Fukuhara**

Japan

**Valerio Fulci**

Italy

**Nicholas Funderburg**

USA

**Nicholas Funderburg**

USA

**Mayuko Furuta**

Japan

**Hala Gabr**

Egypt

**Giovanni Gaeta**

Italy

**Bertino Gaetano**

Italy

**Deni Galileo**

USA

**Martha Patricia Gallegos**

Mexico

**Grazia Galleri**

Italy

**Andrea Galli**

Italy

**Gary Edward Gallick**

USA

**Maria Elsa Gambuzza**

Italy

**Nalini Ganesan**

India

**Can Gao**

China

**Chongfeng Gao**

USA

**Spiros D. Garbis**

UK

**Helena M. Garcia**

Portugal

**Iraia García**

Spain

**Mariana Haydee García Hernández**

Mexico

**Marisa Gariglio**

Italy

**Charlie Garnett-Benson**

USA

**Andrei Gartel**

USA

**Luca Gattinoni**

USA

**Ruowen Ge**

Singapore

**Joel Gelfand**

USA

**Panagiotis Georgianos**

Greece

**Hanspeter Gerber**

USA

**Amato Giaccia**

USA

**Luca Giacomelli**

Italy

**Clemens Giessen**

Germany

**Carla Giordano**

Italy

**Giorgia Girotto**

Italy

**Rebecca Gladdy**

Canada

**W. Scott Goebel**

USA

**Sucheta Gokhale**

India

**Diego Golombek**

Argentina

**Ping Gong**

China

**Ramakrishnan Gopalakrishnan**

USA

**Patricia Grabowski**

Germany

**Stephan Grage**

Germany

**Jennifer Granick**

USA

**Marco Grasso**

Italy

France**sca Grati**

Italy

**Tim Greten**

USA

**Jörg Großhans**

Germany

**Viktor Grünwald**

Germany

**Congying Gu**

USA

**Jian Guan**

China

**Andrea Guennoun**

Germany

**Andrea Guennoun**

Qatar

**Pokhraj Guha**

India

**Maria Valle Guijarro**

USA

**Hongqian Guob**

USA

**Rakesh Gupta**

India

**Raphael Guzman**

Switzerland

**Duy Ha**

USA

**Patrick Ha**

USA

**Ramsey Hachem**

USA

**Abigail Hackam**

USA

**Michael Hackenberg**

Spain

**Monika Haemmerle**

USA

**Weidong Han**

China

**Patrick Hanley**

USA

**Joshua Hare**

USA

**Ulrike Harre**

Germany

**Lauren Harshman**

USA

**Akira Hashiramoto**

Japan

**Larisa Haupt**

Australia

**Yong He**

China

**Nongyue He**

China

**Gen He**

China

**Xiangjun He**

China

**Meian He**

China

**Lionel Hebbard**

Australia

**Doreen Heckmann-Nötzel**

Germany

**Sean Heffron**

USA

**Shelly Heimfeld**

USA

**Mahasti Hemanth**

India

**Peiman Hematti**

USA

**Alonso Heredia**

USA

**Caroline Herron**

Ireland

**Em Higa**

Brazil

**Henry Higgs**

USA

**Jason Hinman**

USA

**Christoph Hoeller**

Austria

**L. John Hoffer**

Canada

**Marion Hofmann-Bowman**

USA

**Stefan Holdenrieder**

Germany

**Arne Homann**

Germany

**Huang Hongbiao**

China

**Risto Honkanen**

Finland

**Xuwei Hou**

China

**Liwen Hu**

China

**Wanchung Hu**

Taiwan

**Guo-Fu Hu**

USA

**Xiaojun Huang**

China

**Chen Huang**

China

**Kuo-How Huang**

Taiwan

**Michael Hudecek**

Germany

**Petra Hudler**

Slovenia

**Kam Hui**

Singapore

**Esther Hulleman**

The Netherlands

**Amanda Hummon**

USA

**Bonnie Hylander**

USA

**Tommaso Iannitti**

UK

**Thomas Ichim**

USA

**Giandomenica Iezzi**

Switzerland

**Imran Imran**

Germany

**Giuseppe Intini**

USA

**Nida Iqbal**

India

**Demelza Ireland**

Australia

**Idilman Is**

Turkey

**Nobukazu Ishizaka**

Japan

**Anna Isinger Ekstrand**

Sweden

**Ayesha Ismail**

India

**Raphael Isokpehi**

USA

**Tomer Itkin**

Israel

**Sawa Ito**

USA

**Keisuke Ito**

USA

**Takahashi J.**

Japan

**Li J.**

China

**Naranamangalam Jagannathan**

India

**Rubio Martinez Jaime**

Spain

**Andreas Janecke**

Austria

**Chaweewan Jansakul**

Thailand

**Jos Jansen**

The Netherlands

**Frank Jenkins**

USA

**Christie Jeon**

USA

**Mark Jeschke**

Canada

**Bente Jespersen**

Denmark

**Bin Ji**

China

**Chen Jiang**

China

**Lili Jiang**

China

**Yanfang Jiang**

China

**Kaiyu Jiang**

USA

**Pengfei Jiang**

USA

**Zhengyu Jiang**

USA

**Amjad Jilani**

UK

**Sergio Jimenez**

USA

**Ying Jin**

China

**Zhiming Jin**

China

**Ping Jin**

USA

**Puthen Jithesh**

UK

**Pascal David Johann**

Germany

**C. Hertzman Johansson**

Sweden

**Andrew Jones**

UK

**Aditya Joshi**

USA

**Oscar Juan**

Spain

**Park Jw**

South Korea

**Kazuhiro Kakimi**

Japan

**Michel Kalamarides**

France

**Kimberly Kalli**

USA

**Steve Kalloger**

Canada

**D. Kalvakolano**

USA

**Masakazu Kamata**

USA

**Nobuhiro Kanaji**

Japan

**Jia-Horng Kao**

Taiwan

**Mariana Kaplan**

USA

**Stylianos Karatapanis**

Greece

**Saoussen Karray**

France

**Mohammed Kashani-Sabet**

USA

**Hiroaki Kataoka**

Japan

**Rahul Kathariya**

India

**Johji Kato**

Japan

**Manabu Kawada**

Japan

**Melissa Kazantzis**

USA

**Andreas Keller**

Germany

**Gottfried Kellermann**

USA

**Christian Kellner**

Germany

**Philippe Kemoun**

France

**Brian Kennedy**

USA

**Robert Kennedy**

USA

**Sid Kerkar**

USA

**Peter Kerkhof**

The Netherlands

**Santosh Kesari**

USA

**Ahmad Khalil**

USA

**Mohammad Khan**

Saudi Arabia

**Mohsin Khan**

USA

**Mushfiquddin Khan**

USA

**Sameer Khandhar**

USA

**Tobias Kiesslich**

Austria

**Bg Kim**

South Korea

**Youngsoo Kim**

South Korea

**Sung-Hwan Kim**

South Korea

**Jang-Hee Kim**

South Korea

**Sung Kim**

South Korea

**Sh Kim**

South Korea

**Ju Kim**

USA

**Chan Hyuk Kim**

USA

**Gaspar Kitange**

USA

**Helmut Klocker**

Austria

**Joost Kluiver**

The Netherlands

**Jared Knickelbein**

USA

**Karl Richard Alexander Knuth**

Qatar

**Mario Kofler**

Austria

**Feliks Kogan**

USA

**Takashi Kohno**

Japan

**Navid Koleini**

Canada

**Tomas Konecny**

USA

**Gordana Konjevic**

Serbia

**Izabela Korczowska**

Poland

**Srigiridhar Kotamraju**

India

**Nir Krakauer**

USA

**Oliver Holger Krämer**

Germany

**Stephanie Kreis**

Luxembourg

**Prasanna Krishnamurthy**

USA

**Karl Krueger**

USA

**Kostyantyn Krysan**

USA

**Dominika Ksiazek-Winiarek**

Poland

**Chia-Yi Kuan**

USA

**Selim Kuci**

Germany

**Ingo Kuhfuss**

Germany

**Joydeb Kumar Kundu**

Bangladesh

**Kv O’brien**

USA

**Peter Kwong**

USA

**Maris Laan**

Estonia

**Ahmed Labib**

Qatar

**Moncef Ladjimi**

France

**Brian Lane**

USA

**Philip Lang**

Germany

France**sca Lantieri**

Italy

**Jacob Larkin**

USA

**Göran Larson**

Sweden

**Juan Jose Lasarte**

Spain

**George Lau**

Hong Kong

**Andrew Leask**

Canada

**Francis Y. Lee**

USA

**Hye Seung Lee**

Hye2@Snu.Ac.Kr

**Justine Lee**

USA

**Kyoo-Hyung Lee**

South Korea

**Hsin-Chen Lee**

Taiwan

**Linsheng Lei**

China

**Raya Leibowitz-Amit**

USA

**Mathieu Lemaire**

Canada

**Gabor Lendvai**

Hungary

**Nibedita Lenka**

India

**Leticia Leon**

Spain

**Eleonora Leucci**

Belgium

**Estrella Levy**

Argentina

**Alan Lewis**

USA

**Alan Lewis**

USA

**Zhijie Li**

Australia

**Yan Li**

China

**Pu Li**

China

**Jinjun Li**

China

**Leping Li**

China

**Bing Li**

China

**Zhong Li**

China

**Chenggang Li**

China

**Zhe Li**

USA

**Qing Kay Li**

USA

**Chih-Chuang Liaw**

Taiwan

**Chuyong Lin**

China

**Jian Lin**

China

**Chao-Hsiung Lin**

Taiwan

**Li-Min Lin**

Taiwan

**Sue-Hwa Lin**

USA

**David Lin**

USA

**Jere Linden**

Finland

**Yinghui Ling**

China

**Lance Liotta**

USA

**Lei Liu**

China

**Song-Mei Liu**

China

**Shaorong Liu**

USA

**Zhaohui Liu**

China

**Víctor Llombart**

Spain

**David M. Loeb**

USA

**Markus Loeffler**

Germany

**Vincent Lombardi**

USA

**Lucia Lopalco**

Italy

**Elena Lopez Villar**

Spain

**César López-Camarillo**

Mexico

**Antonio Lopez-Sanroman**

Spain

**Marek Łos**

Sweden

**Jennifer Lowe**

Brazil

**Jiamiao Lu**

USA

**Xiongbin Lu**

USA

**Guilherme Lucas**

Brazil

**Tim Luckett**

Australia

**Jana Luetzkendorf**

Germany

**Enrico Lugli**

Italy

**Miodrag Lukic**

Serbia

**Ns Lurain**

USA

**Hongjun Lv**

China

**Anastasios Lymperopoulos**

USA

**Duan Ma**

China

**Daoxin Ma**

China

**Muzafar Ahmad Macha**

USA

**Catarina Madeira**

Portugal

**Margaret Madeleine**

USA

**Roberta Maggio**

Italy

**Karl-Eric Magnusson**

Sweden

**Kathleen Mahoney**

USA

**Kristen Maitland**

USA

**Eugene Major**

USA

**Joel Malek**

Qatar

**Mauro Malnati**

Italy

**Masaki Mandai**

Japan

**Stuart Mannering**

Australia

**Bobbak Mansouri**

USA

**Ashham Mansur**

Germany

**Yumeng Mao**

Sweden

**Armen Mardiros**

USA

**Annette Mareau**

USA

**Katia Mareschi**

Italy

**Paula Marincola**

USA

**Maria Marino**

Italy

**Andrea Markus**

Germany

**Giuseppe Marraro**

Italy

**Gian Luigi Marseglia**

Italy

**Francis Martin**

UK

**Daniel Martins-De-Souza**

Germany

**Tetsuo Maruyama**

Japan

**Emanuela Masini**

Italy

**Andrea Masotti**

Italy

**Giuseppe Masucci**

Sweden

**António Mata**

Portugal

**Jesús Mateos**

Spain

**Alicia Mathers**

USA

**Scot Matkovich**

USA

**Satoaki Matoba**

Japan

**Konstantinos Mavridis**

Greece

**Rose-Anne Mazure**

Spain

**Guillermo Mazzolini**

Argentina

**Kerrie Mcdonald**

Australia

**Donald Mcdonnell**

USA

**David Mckenna**

USA

**Randall Mckenna**

USA

**Radwa Mehanna**

Egypt

**Chitra Mehta**

India

**Shu Meng**

China

**Pier Luigi Meroni**

Italy

**Claudio Mesquita**

Brazil

**Patrick Meybohm**

Germany

**Thomas Meyer**

Germany

**Raposo Mf.**

Portugal

**Giancarlo Micheletto**

Italy

**Miguel Miguens**

Spain

**Michal Mikula**

Poland

**Humberto Milani**

Brazil

**Leslie Miller**

USA

**Wei-Ping Min**

Canada

**Silvia Minardi**

USA

**Boris Minev**

USA

**B. Minev**

USA

**Boris Minev**

USA

**Amadeo Minichino**

Italy

**Eva M. Mischak-Weissinger**

Germany

**Tom Misteli**

USA

**Ashim K. Mitra**

USA

**Tomomitsu Miyagaki**

Japan

**Yasuyoshi Miyata**

Japan

**Hiroshi Miyata**

Japan

**Alessandra Mocali**

Italy

**Marius Moga**

Romania

**Geetha Mohan**

USA

**Suyash Mohan**

USA

**Rajesh Mohanraj**

United Arab Emirates

**Giuseppe Montalto**

Italy

**Bo-Hyun Moon**

USA

**Martin Moore**

USA

**Ana Maria Mora**

Costa Rica

**Silvana Morello**

Italy

**Richard Morgan**

USA

**Don Morris**

Canada

**Giovanni Morrone**

Italy

**Mohamed Morsy**

Saudi Arabia

**Seyed Javad Mowla**

Iran

**Arijit Mukhopadhyay**

India

**Debabrata Mukhopadhyay**

USA

**Yosuke Mukoyama**

USA

**Dafne Müller**

Germany

**Carine Munaut**

Belgium

**Susan Murphy**

USA

**William Murphy**

USA

**Teemu Murtola**

Finland

**Saminathan Muthusamy**

USA

**Seyed Mohammad Nabavi**

Iran

**Takeshi Nabe**

Japan

**Atsushi Nagai**

Japan

**S. Nagini**

India

**Takayuki Nakagawa**

Japan

**Yoshikazu Nakamura**

Japan

**Kazuhiro Nakayama**

Japan

**Manouchehr Nakhjavani**

Iran

**Marek Nalos**

Australia

**Norbert Nass**

Germany

**Pier Giorgio Natali**

Italy

**Urs Nater**

Germany

**Pilar Navarro**

Spain

**Marcos Mônico Neto**

USA

**Qimin Ng**

USA

**Robert Nibbs**

UK

**Michael Nicholson**

UK

**Amelia Nieto**

Spain

**Hataikarn Nimitphong**

Thailand

**Paola Nistico**

Italy

**Zhaoshan Niu**

China

**Paul Norris**

USA

**Andrew Norris**

USA

**Mauro Novelli**

Italy

**Gannon Np**

USA

**Heather M. O’hagan**

USA

**Natasa Obermajer**

USA

**J O’callaghan**

UK

**Amanda Ode**

Sweden

**Pardo Oe**

UK

**Mitsuru Ohishi**

Japan

**Shigeo Ohta**

Japan

**Masato Okamoto**

Japan

**H. Okazawa**

Japan

**José M. Olmos**

Spain

**Oluwadayo Oluwadara**

USA

**Petr Ostadal**

Czech Republic

**Arne Ostman**

Sweden

**Megan Othus**

USA

**Christian Ottensmeier**

UK

**Teresa Otto**

Germany

**C. Keith Ozaki**

USA

**Utpal Pal**

USA

**Nallasivam Palanisamy**

USA

**Michael Palladino**

USA

**Eleonora Palma**

Italy

**Gaby Palmer**

Switzerland

**Giuseppe Palmieri**

Italy

**Xinmin Pan**

China

**Sandhya Panch**

USA

**Tanmay Panchabhai**

USA

**Jong Hoon Park**

South Korea

**Jiyoung Park**

South Korea

**Mr. Parkhurst**

USA

**Paola Parronchi**

Italy

**Rosa Maria Pascale**

Italy

**Shibani Pati**

USA

**Bhushan Patwardhan**

India

**Andreas Paul**

Germany

**Kajsa Maria Paulsson**

Sweden

**Henrik D. Pedersen**

Denmark

**Xue Tao Pei**

China

**Maria Marjorette Pena**

USA

**Dao-Quan Peng**

China

**Cheng-Yuan Peng**

Taiwan, China

**Arianne Perez Garcia**

USA

**Sven Perner**

Germany

**Carlo Perricone**

Italy

**Michael Peters**

UK

**Bryon Petersen**

USA

**Annacarmen Petrizzo**

Italy

**Raffaele Pezzilli**

Italy

**Matthew Phillips**

USA

**Giulia Piaggio**

Italy

**Massimo Pietropaolo**

USA

**Valeria Pietropaolo**

Italy

**Somchai Pinlaor**

Thailand

**Karina Pino-Lagos**

Chile

**Jalil Pirayesh Islamian**

Iran

**Anna Planas**

Spain

**Martin Playford**

USA

**Miroslav Pohanka**

Czech Republic

**Raimo Pohjanvirta**

Finland

**Katherine Ponder**

USA

**Zoltán Pós**

Hungary

**Michael Postow**

USA

**David Pride**

USA

**Maurizio Provenzano**

Switzerland

**Igor Prudovsky**

Russian Federation

**Ioannis Psallidas**

UK

**Michele Purrello**

Italy

**Alessandro Puzziello**

Italy

**Hui Qian**

China

**Fabiana Quaglia**

Italy

**Mohammed Quttainah**

Saudi Arabia

**Monte Radeke**

USA

**Gajendra Raghava**

India

**Afsar Rahbar**

Sweden

**Natalia Ramirez**

Spain

**Famela Ramos**

USA

**Kanishka Ratnayaka**

USA

**Arnab Ray**

USA

**Elrashdy Redwan**

Saudi Arabia

**Kerstin Reimers**

Germany

**Jiaqiang Ren**

USA

**Anna Lena Ress**

Austria

**Habtom Ressom**

USA

**Muhammad Riaz**

Pakistan

**Andrea Ribeiro-Dos-Santos**

Brazil

**Biagio Ricciuti**

Italy

**Ann Richmond**

USA

**Neil Riordan**

Panama

**Loris Rizzello**

UK

**Lidia Robert**

USA

**Anna Rocco**

Italy

**Rafael Rocha**

Brazil

**Ramón Rodrigo**

Chile

**Rosalia Rodriguez–Rodriguez**

Spain

**Howard Roemer**

USA

**Michael Rogers**

USA

**Andreas Roidl**

Germany

**Francisco Romero**

Spain

**Dario Ronchi**

Italy

**Guanghua Rong**

China

**Ronald Rooke**

France

**Margaret Ross**

USA

**Giulio Rossi**

Italy

**Claudia Rossig**

Germany

**Susanta Roychoudhury**

India

**Paolo Ruggiero**

Italy

**Pekka Ruotsalainen**

Finland

**Julie Russier**

France

**Sergio Rutella**

Qatar

**Sergio Rutella**

Qatar

**Carrie Ruxton**

UK

**Ogino S.**

USA

**Bashar Saad**

Palestinian Territory

**Marianna Sabatino**

USA

**Ulrich Sack**

Germany

**Sakthivel Sadayappan**

USA

**Sona Saksena**

USA

**Gianluca Sala**

Italy

**Athanasios Salifoglou**

Greece

**Gil Salles**

Brazil

**Goli Samimi**

USA

**Marta Sanchez-Carbayo**

Spain

**Pau Sancho-Bru**

Spain

**Francesca Santoni De Sio**

Italy

**Ney Santos**

Brazil

**Gaetano Santulli**

USA

**Anna Sapino**

Italy

**Shantanu Sapru**

India

**Francesca Sarlo**

Italy

**Manoj Sarma**

USA

**Seetharama Sastry Konduru**

Qatar

**Jean Frederic Sauniere**

France

**Nazish Sayed**

USA

**Giorgio Scagliotti**

Italy

**Richard Schaefer**

Germany

**Francesco Paolo Schena**

Italy

**Howard Scher**

USA

**Sophia E. Schiza**

Greece

**Erwin Schleicher**

Germany

**Dorit Schleinitz**

Germany

**Antje Schmidt**

Germany

**Gerold Schuler**

Germany

**Sandra Schüssler**

Austria

**Clare Scott**

Australia

**Cristian Secchi**

Italy

**Silvia Selleri**

Canada

**Carlo Selmi**

USA

**Lorenzo Sempere**

USA

**Aritro Sen**

USA

**Nabonita Sengupta**

India

**Sung Wook Seo**

USA

**Naresh Babu V. Sepuri**

India

**Suraj Serai**

USA

**Natalya Seredkina**

Norway

**Feride Severcan**

Turkey

**Patricia Severino**

Brazil

**Ioannis Sgouralis**

USA

**Ramraj Sh.**

USA

**Baoen Shan**

China

**Jingxuan Shan**

Qatar

**E. Sharples**

UK

**Yuehwei Shen**

USA

**Rui Sheng**

China

**Natalie Shenker**

UK

**Hilary Sheppard**

New Zealand

**Warren Sherman**

Belgium

**Yongguo Shi**

China

**Ruihua Shi**

China

**Lin Shi**

China

**Ning Shi**

USA

**Takatsune Shimizu**

Japan

**Yoshihiro Shirai**

Japan

**Yehuda Shoenfeld**

Israel

**Emma Shtivelman**

USA

**Thomas Shupe**

USA

**Bruno Silva-Santos**

Portugal

**Charles Simone**

USA

**James Simpkins**

USA

**Peter Sims**

USA

**Virendra Singh**

India

**Anup Singh**

India

**Ritesh Singh**

India

**Arvind Singla**

Canada

**Sridhar Sivasubbu**

India

**Tomas Skripcak**

Germany

**Philipp Skroblin**

UK

**Nicoline Smit**

The Netherlands

**Maarten Snoeijs**

The Netherlands

**Robert Sobol**

USA

**Joyce Solheim**

USA

**David Solomon**

USA

**Mark Soloski**

USA

**Michele Sommariva**

Italy

**Deok-Soo Son**

USA

**Avinash Sonawane**

India

**Libing Song**

China

**Koh-Hei Sonoda**

Japan

**Claudio Sorio**

Italy

**Alexander Sorokin**

USA

**Vinicius Sortica**

Brazil

**John Souglakos**

Greece

**Andrew South**

USA

**Claudio Spada**

Italy

**Giulio Spagnoli**

Switzerland

**Christoph Spiess**

USA

**Neil J. Spratt**

Australia

**Ralf Sprengers**

The Netherlands

**Danielle Springer**

USA

**Randall Stafford**

Canada

**Robert Stahelin**

USA

**Ivan Stanojevic**

Serbia

**Elena Stansky**

USA

**Ted Steiner**

Canada

**Marta Stelmach-Mardas**

Germany

**Verena Stiegelbauer**

Austria

**Georg Stoecklin**

Germany

**Karen Stokes**

USA

**Jan Stolk**

The Netherlands

**Areti Strati**

Greece

**Raphael Stricker**

USA

**Patrick Stuart**

USA

**Christina Stuelten**

USA

**Ali Sultan**

USA

**Shubhankar Suman**

USA

**Ming Sun**

China

**Huijun Sun**

China

**Tao Sun**

USA

**Yang Sun**

USA

**Inge Marie Svane**

Denmark

**Abhisek Swaika**

USA

**Wing-Kin Syn**

UK

**Sébastien Tabariès**

Canada

**Frank Tacke**

Germany

**Cliff Taggart**

UK

**Maria Tagliamonte**

Italy

**Hiroshi Takemori**

Japan

**Daryush Talei**

Iran

**Giuseppe Tambussi**

Italy

**Yi Tan**

USA

**Eng M. Tan**

USA

**Xianglin Tan**

USA

**Takuji Tanaka**

Japan

**Halil Tanboga**

Turkey

**Qionglan Tang**

China

**Jing Tang**

China

**Jianming Tang**

USA

**Yaoliang Tang**

USA

**Magdalena Tary-Lehmann**

USA

**Francesca Tavano**

Italy

**Kathryn Taylor**

UK

**Heather Teague**

USA

**Mohamed-Ramzi Temanni**

Qatar

**Anja Ten Brinke**

The Netherlands

**Benno Ter Kuile**

The Netherlands

**Yasuo Terauchi**

Japan

**Luigi Terracciano**

Switzerland

**Paula Terraciano**

Brazil

**Annalisa Terranegra**

Italy

**Annalisa Terranegra**

Qatar

**Osamu Tetsu**

USA

**Shikha Tewari**

India

**Charles Theuer**

USA

**Susan Thibeault**

USA

**Gaetano Thiene**

Italy

**Victor Ljl Thijssen**

The Netherlands

**Albert Thomas**

USA

**M. Albert Thomas**

USA

**Bolaji Thomas**

USA

**Tanushree Thote**

USA

**Weidong Tian**

China

**Jozsef Timar**

Hungary

**Vincent Timmerman**

Belgium

**Antony Tin**

USA

**Marianne Tinguely**

Switzerland

**Gabriele Toietta**

Italy

**Maria Lauda Tomasi**

USA

**Sara Tomei**

Qatar

**Maja Tomicic**

Germany

**Ikuo Tooyama**

Japan

**Maria Lina Tornesello**

Italy

**Gregory Tranah**

USA

**Gabriela Trevisan**

Brazil

**Hui-Ju Tsai**

Taiwan, China

**John Tsang**

USA

**Marco Tucci**

Italy

**Paul Tumeh**

USA

**Tao-Hsin Tung**

Taiwan

**Heth Turnquist**

USA

**Cameron Turtle**

USA

**Sandra Tuyaerts**

Belgium

**Argyris Tzouvelekis**

Greece

**Naoto Ueno**

USA

**Lena Uller**

Sweden

**Cuneyt Ulutin**

Turkey

**Florian Unger**

Germany

**Masuko Ushio-Fukai**

USA

**Pascal Vachon**

Canada

**Silvia Vai**

Italy

**Mariapia Vairetti**

Italy

**Sergey Y. Vakhrushev**

Denmark

**Soraya Valles**

Spain

**Johan Van Der Vlag**

The Netherlands

**Eric Van Dyck**

Luxembourg

**Mireille Van Gele**

Belgium

**Arthur Vandenbark**

USA

**Moriel Vandsburger**

USA

**Venkat Vangaveti**

Australia

**Carlos Vasconcelos**

Portugal

**Roger Vaughan**

USA

**Florence Velge-Roussel**

France

**Paulo Verardi**

USA

**Mohamud Verjee**

Qatar

**Suresh Verma**

USA

**Gianpaolo Vidili**

Italy

**Regina Maria Vilela**

Brazil

**Sandhya Visweswariah**

India

**Flavia Vitale**

USA

**Santiago Viteri**

Spain

**Isabel Vogel**

France

**Barbara Vona**

Germany

**Agarwal Vr.**

India

**Lukas Vrba**

USA

**Krishna Vyas**

USA

**Ori Wald**

Israel

**David Walker**

Austria

**Judd Walson**

USA

**Yu-Jui Wan**

USA

**Hans Wandall**

Denmark

**Yingwei Wang**

China

**Haiyun Wang**

China

**Anhui Wang**

China

**Xin Wang**

China

**Shukui Wang**

China

**De-Shen Wang**

China

**Jun Wang**

China

**Feng-Sheng Wang**

Taiwan

**Shih-Wei Wang**

Taiwan

**Weiguang Wang**

UK

**I-Ming Wang**

USA

**Zhaoming Wang**

USA

**Chunling Wang**

China

**Rui Wang-Sattler**

Germany

**Yoo Wan-Hee**

South Korea

**Kelly Warfield**

USA

**David G. Watt**

UK

**Jeffrey Weber**

USA

**Barbara Wegiel**

USA

**Qize Wei**

USA

**George Weiner**

USA

**Herschel Weintraub**

USA

**James Wells**

Australia

**James Welsh**

USA

**Tom Van Wezel**

The Netherlands

**Nicole White**

USA

**Matt Whiteman**

UK

**Robert Wilensky**

USA

**Karen Willard-Gallo**

Belgium

**Jed Wiltzius**

USA

**Johann Wojta**

Austria

**Chris Wong**

China

**Thian-Sze Wong**

Hong Kong

**Wing Tak Jack Wong**

USA

**Haijing Wu**

China

**Yingli Wu**

China

**Weizhong Wu**

China

**Wen-Bin Wu**

Taiwan

**Reen Wu**

USA

**Xiaobo Wu**

USA

**Chia-Chao Wu**

Taiwan

**Vegard Bruun Wyller**

Norway

**Mo X.**

China

**Zhidao Xia**

UK

**Andy Xiang**

China

**Zhengwen Xiao**

Canada

**Hang Xiao**

China

**G. Xie**

China

**Baoling Xing**

China

**Feng Xu**

China

**Rui-Hua Xu**

China

**Wenrong Xu**

China

**Feng Xu**

China

**Sushma Yadav**

USA

**Feng Yan**

China

**Jun Yan**

China

**Maria Yanez-Mo**

Spain

**Qinglin Yang**

China

**Zhixiong Yang**

China

**Cheng Yang**

China

**Kejian Yang**

USA

**Wassana Yantasee**

USA

**Jiayi Yao**

China

**Ming Yao**

China

**Kosei Yasumoto**

Japan

**Dongqing Ye**

China

**Shoudong Ye**

China

**Yao-Tsung Yeh**

Taiwan

**Samantha Yeligar**

USA

**Pen-Hui Yin**

Taiwan

**Song Yongmei**

China

**Yukie Yoshii**

Japan

**Seungkwon You**

South Korea

**Jianxiu Yu**

China

**Tao Yu**

China

**Hao Yu**

USA

**Jean-François Zagury**

France

**Marco Zarbin**

USA

**Paul Zarogoulidis**

Greece

**Amir Zarrinpar**

USA

**Flora Zavala**

France

**Abdel-Rahman Zekri**

Egypt

**Davide Zella**

USA

**Dimitrios Zeugolis**

Ireland

**Shu Zhang**

China

**He Zhang**

China

**Shenghui Zhang**

China

**Di Zhang**

China

**Haitao Zhao**

China

**Ming Zhao**

China

**Dejian Zhao**

China

**Minghui Zhao**

China

**Junhua Zheng**

China

**Fei Zhou**

China

**Yunfeng Zhou**

China

**J. Zhou**

China

**Xi Kathy Zhou**

USA

**Dengna Zhu**

China

**Ping Zhu**

China

**Shaobo Zhu**

USA

**David Zidar**

USA

**Anna Linda Zignego**

Italy

